# Supplementary material for: Yiai Fuzheng decoction inhibits triple-negative breast cancer by remodeling the immune microenvironment
Source: Front Immunol. 2025 Sep 30;16:1615631. doi: 10.3389/fimmu.2025.1615631 (PMC12518410; doi:10.3389/fimmu.2025.1615631)
Supplement: Supplementary file 2 [file DataSheet1.zip › Supplementary File 2/Treatment/C_vs_D_vs_H/06_Pathway_Analysis/Pathway_Analysis/Sig_Pathways_Name/Purine metabolism.pdf]

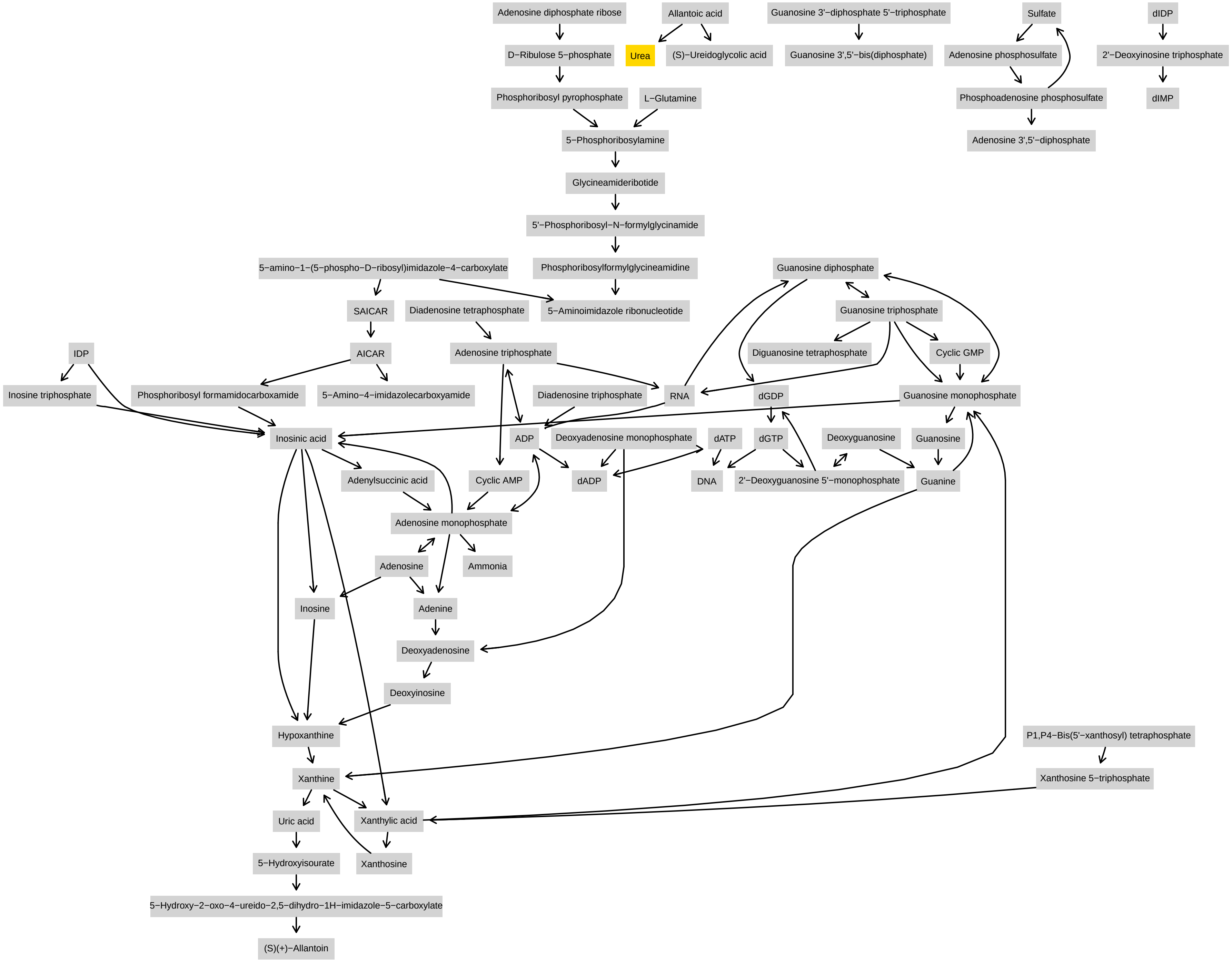
Adenosine diphosphate ribose
Allantoic acid
Guanosine 3'−diphosphate 5'−triphosphate
Sulfate
dIDP
D−Ribulose 5−phosphate
Urea
(S)−Ureidoglycolic acid
Guanosine 3',5'−bis(diphosphate)
Adenosine phosphosulfate
2'−Deoxyinosine triphosphate
Phosphoribosyl pyrophosphate
L−Glutamine
Phosphoadenosine phosphosulfate
dIMP
5−Phosphoribosylamine
Adenosine 3',5'−diphosphate
Glycineamideribotide
5'−Phosphoribosyl−N−formylglycinamide
5−amino−1−(5−phospho−D−ribosyl)imidazole−4−carboxylate
Phosphoribosylformylglycineamidine
Guanosine diphosphate
SAICAR
Diadenosine tetraphosphate
5−Aminoimidazole ribonucleotide
Guanosine triphosphate
IDP
AICAR
Adenosine triphosphate
Diguanosine tetraphosphate
Cyclic GMP
Inosine triphosphate
Phosphoribosyl formamidocarboxamide
5−Amino−4−imidazolecarboxyamide
Diadenosine triphosphate
RNA
dGDP
Guanosine monophosphate
Inosinic acid
ADP
Deoxyadenosine monophosphate
dATP
dGTP
Deoxyguanosine
Guanosine
Adenylsuccinic acid
Cyclic AMP
dADP
DNA
2'−Deoxyguanosine 5'−monophosphate
Guanine
Adenosine monophosphate
Adenosine
Ammonia
Inosine
Adenine
Deoxyadenosine
Deoxyinosine
Hypoxanthine
P1,P4−Bis(5'−xanthosyl) tetraphosphate
Xanthine
Xanthosine 5−triphosphate
Uric acid
Xanthylic acid
5−Hydroxyisourate
Xanthosine
5−Hydroxy−2−oxo−4−ureido−2,5−dihydro−1H−imidazole−5−carboxylate
(S)(+)−Allantoin
